# 4-(Hydroxy­meth­yl)phenol

**DOI:** 10.1107/S1600536809022466

**Published:** 2009-06-27

**Authors:** Wei-Sheng Liu, Rui-Ping Wei, Xiao-Liang Tang, Wen-Hua Wang, Zheng-Hua Ju

**Affiliations:** a Department of Chemistry, State Key Laboratory of Applied Organic Chemistry, College of Chemical Engineering, Lanzhou University, Lanzhou 730000, People’s Republic of China; b State Key Laboratory of Coordination Chemistry, Nanjing University, Nanjing 210093, People’s Republic of China; c College of Chemistry and Chemical Engineering and State Key Laboratory of Applied Organic Chemistry, Lanzhou University, Lanzhou 730000, People’s Republic of China

## Abstract

In the mol­ecule of the title compound, C_7_H_8_O_2_, the phenol O and hydroxy­methyl C atoms lie in the ring plane [deviations of −0.015 (3) and and 0.013 (3) Å, respectively]. In the crystal structure, inter­molecular O—H⋯O hydrogen bonds link mol­ecules into a network. A weak C—H⋯π inter­action is also found.

## Related literature

For a related structure, see: Tale *et al.* (2003 ▶). For bond-length data, see: Allen *et al.* (1987 ▶).
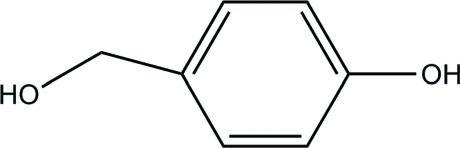


## Experimental

### 

#### Crystal data

C_7_H_8_O_2_
*M* *_r_* = 124.13
Orthorhombic, 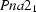

*a* = 9.524 (3) Å
*b* = 11.006 (4) Å
*c* = 5.942 (2) Å
*V* = 622.9 (4) Å^3^
*Z* = 4
Mo *K*α radiation
μ = 0.10 mm^−1^
*T* = 298 K
0.65 × 0.62 × 0.55 mm

#### Data collection

Bruker SMART CCD area-detector diffractometer
Absorption correction: multi-scan (*SADABS*; Sheldrick, 1996 ▶) *T*_min_ = 0.940, *T*_max_ = 0.949
3751 measured reflections
1414 independent reflections
1200 reflections with *I* > 2σ(*I*)
*R*_int_ = 0.036

#### Refinement

*R*[*F* ^2^ > 2σ(*F* ^2^)] = 0.035
*wR*(*F* ^2^) = 0.076
*S* = 1.00
1414 reflections
84 parameters
1 restraint
H-atom parameters constrained
Δρ_max_ = 0.15 e Å^−3^
Δρ_min_ = −0.17 e Å^−3^

### 

Data collection: *SMART* (Bruker, 2000 ▶); cell refinement: *SAINT* (Bruker, 2000 ▶); data reduction: *SAINT*; program(s) used to solve structure: *SHELXS97* (Sheldrick, 2008 ▶); program(s) used to refine structure: *SHELXL97* (Sheldrick, 2008 ▶); molecular graphics: *SHELXTL* (Sheldrick, 2008 ▶) and *PLATON* (Spek, 2009 ▶); software used to prepare material for publication: *SHELXTL* and *PLATON*.

## Supplementary Material

Crystal structure: contains datablocks I, global. DOI: 10.1107/S1600536809022466/hk2691sup1.cif

Structure factors: contains datablocks I. DOI: 10.1107/S1600536809022466/hk2691Isup2.hkl

Additional supplementary materials:  crystallographic information; 3D view; checkCIF report

## Figures and Tables

**Table 1:** Hydrogen-bond geometry (Å, °)

*D*—H⋯*A*	*D*—H	H⋯*A*	*D*⋯*A*	*D*—H⋯*A*
O1—H1⋯O2^i^	0.82	1.86	2.668 (3)	169
O2—H2⋯O1^ii^	0.82	2.01	2.817 (3)	167
C1—H1*B*⋯*Cg*1^iii^	0.97	2.77	3.694 (3)	159

Symmetry codes: (i) 
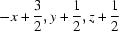
; (ii) 
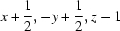
; (iii) 
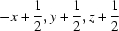
. *Cg*1 is the centroid of the C2–C7 ring.

**Fig. 1.**
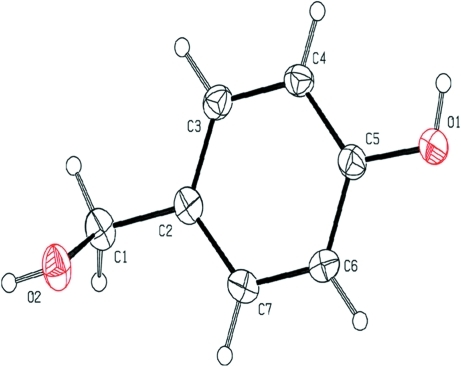
The molecular structure of the title molecule, with the atom-numbering scheme. Displacement ellipsoids are drawn at the 30% probability level.

**Fig. 2.**
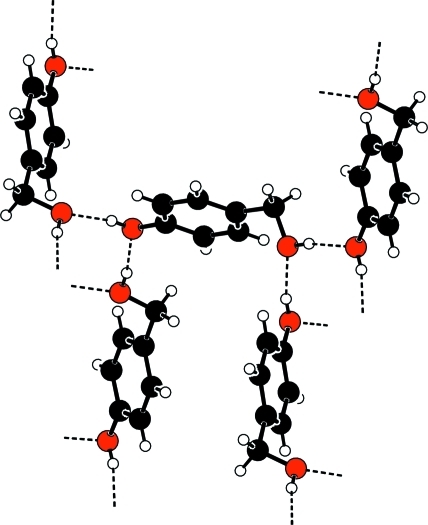
A partial packing diagram of the title compound. Hydrogen bonds are shown as dashed lines.

## supplementary crystallographic information

### Comment

The reduction of carboxylic acids to alcohols is a key synthetic transformation in organic chemistry. There are several ways to bring about this transformation. It is conventionally carried out using sodium borohydride as a reducing agent. We report herein the crystal structure of the title compound.

In the molecule of the title compound (Fig 1), the bond lengths (Allen *et al.*,  1987) and angles are within normal ranges. Ring A (C2-C7) is, of course, planar. Atoms O1, O2 and C1 are -0.015 (3), 1.279 (3) and 0.013 (3) Å away from the ring plane, respectively.

In the crystal structure, intermolecular O-H···O hydrogen bonds (Table 1) link the molecules into a network, in which they may be effective in the stabilization of the structure. There also exists a weak C—H···π interaction (Table 1).

### Experimental

The title compound was prepared by reducing corresponding carboxylic acid using sodium borohydride in THF solution according to a literatue method (Tale *et al.*,  2003). Crystals suitable for X-ray analysis were obtained by slow evaporation of an ethyl acetate solution.

### Refinement

H atoms were positioned geometrically, with O-H = 0.82 Å (for OH) and C-H = 0.93 and 0.97 Å for aromatic and methylene H, respectively, and constrained to ride on their parent atoms, with U_iso_(H) = xU_eq_(C,O), where x = 1.5 for OH H and x = 1.2 for all other H atoms. The absolute structure could not be determined reliably, and 605 Friedel pairs were averaged before the last cycle of refinement.

### Crystal data

**Table:**

C_7_H_8_O_2_	*F*(000) = 264
*M**_r_* = 124.13	*D*_x_ = 1.324 Mg m^−^^3^
Orthorhombic, *P**n**a*2_1_	Mo *K*α radiation, λ = 0.71073 Å
Hall symbol: P 2c -2n	Cell parameters from 1897 reflections
*a* = 9.524 (3) Å	θ = 2.8–27.9°
*b* = 11.006 (4) Å	µ = 0.10 mm^−^^1^
*c* = 5.942 (2) Å	*T* = 298 K
*V* = 622.9 (4) Å^3^	Block, colorless
*Z* = 4	0.65 × 0.62 × 0.55 mm

### Data collection

**Table:**

Bruker SMART CCD area-detector diffractometer	1414 independent reflections
Radiation source: fine-focus sealed tube	1200 reflections with *I* > 2σ(*I*)
graphite	*R*_int_ = 0.036
φ and ω scans	θ_max_ = 27.8°, θ_min_ = 2.8°
Absorption correction: multi-scan (*SADABS*; Sheldrick, 1996)	*h* = −12→8
*T*_min_ = 0.940, *T*_max_ = 0.949	*k* = −14→14
3751 measured reflections	*l* = −7→7

### Refinement

**Table:**

Refinement on *F*^2^	Primary atom site location: structure-invariant direct methods
Least-squares matrix: full	Secondary atom site location: difference Fourier map
*R*[*F*^2^ > 2σ(*F*^2^)] = 0.035	Hydrogen site location: inferred from neighbouring sites
*wR*(*F*^2^) = 0.076	H-atom parameters constrained
*S* = 1.00	*w* = 1/[σ^2^(*F*_o_^2^) + (0.0125*P*)^2^ + 0.2042*P*] where *P* = (*F*_o_^2^ + 2*F*_c_^2^)/3
1414 reflections	(Δ/σ)_max_ < 0.001
84 parameters	Δρ_max_ = 0.15 e Å^−^^3^
1 restraint	Δρ_min_ = −0.16 e Å^−^^3^

### Special details

**Table:**

Geometry. All e.s.d.'s (except the e.s.d. in the dihedral angle between two l.s. planes) are estimated using the full covariance matrix. The cell e.s.d.'s are taken into account individually in the estimation of e.s.d.'s in distances, angles and torsion angles; correlations between e.s.d.'s in cell parameters are only used when they are defined by crystal symmetry. An approximate (isotropic) treatment of cell e.s.d.'s is used for estimating e.s.d.'s involving l.s. planes.
Refinement. Refinement of *F*^2^ against ALL reflections. The weighted *R*-factor *wR* and goodness of fit *S* are based on *F*^2^, conventional *R*-factors *R* are based on *F*, with *F* set to zero for negative *F*^2^. The threshold expression of *F*^2^ > σ(*F*^2^) is used only for calculating *R*-factors(gt) *etc*. and is not relevant to the choice of reflections for refinement. *R*-factors based on *F*^2^ are statistically about twice as large as those based on *F*, and *R*- factors based on ALL data will be even larger.

### Fractional atomic coordinates and isotropic or equivalent isotropic displacement parameters (Å^2^)

**Table:**

	*x*	*y*	*z*	*U*_iso_*/*U*_eq_	
O1	0.65915 (14)	0.47478 (11)	0.5585 (2)	0.0477 (3)	
H1	0.6206	0.5344	0.5036	0.072*	
O2	0.99603 (14)	0.15137 (10)	−0.1224 (3)	0.0470 (3)	
H2	1.0542	0.1183	−0.2037	0.071*	
C1	1.05125 (18)	0.26412 (15)	−0.0417 (3)	0.0422 (4)	
H1A	1.0696	0.3180	−0.1675	0.051*	
H1B	1.1391	0.2500	0.0366	0.051*	
C2	0.94754 (17)	0.32168 (14)	0.1149 (3)	0.0351 (4)	
C3	0.87551 (17)	0.42678 (14)	0.0552 (3)	0.0367 (4)	
H3	0.8929	0.4626	−0.0838	0.044*	
C4	0.77818 (18)	0.47925 (15)	0.1993 (3)	0.0356 (4)	
H4	0.7304	0.5492	0.1563	0.043*	
C5	0.75251 (16)	0.42727 (13)	0.4065 (3)	0.0349 (4)	
C6	0.82324 (18)	0.32200 (15)	0.4694 (3)	0.0406 (4)	
H6	0.8058	0.2864	0.6086	0.049*	
C7	0.91947 (18)	0.27084 (15)	0.3239 (3)	0.0411 (4)	
H7	0.9666	0.2006	0.3668	0.049*	

### Atomic displacement parameters (Å^2^)

**Table:**

	*U*^11^	*U*^22^	*U*^33^	*U*^12^	*U*^13^	*U*^23^
O1	0.0571 (8)	0.0424 (7)	0.0437 (8)	0.0143 (6)	0.0121 (7)	0.0079 (6)
O2	0.0487 (7)	0.0391 (6)	0.0532 (8)	−0.0069 (6)	0.0148 (6)	−0.0085 (6)
C1	0.0361 (9)	0.0379 (8)	0.0526 (11)	−0.0041 (7)	0.0067 (8)	−0.0032 (9)
C2	0.0326 (8)	0.0322 (8)	0.0404 (10)	−0.0045 (7)	−0.0004 (7)	−0.0027 (7)
C3	0.0432 (9)	0.0326 (8)	0.0345 (9)	−0.0051 (7)	0.0014 (8)	0.0037 (8)
C4	0.0412 (9)	0.0277 (7)	0.0381 (10)	0.0003 (7)	−0.0028 (8)	0.0030 (7)
C5	0.0365 (8)	0.0308 (7)	0.0374 (9)	0.0000 (6)	−0.0008 (7)	−0.0008 (7)
C6	0.0475 (10)	0.0382 (9)	0.0362 (9)	0.0035 (8)	0.0012 (8)	0.0088 (7)
C7	0.0425 (9)	0.0353 (8)	0.0454 (10)	0.0079 (7)	−0.0023 (8)	0.0035 (8)

### Geometric parameters (Å, °)

**Table:**

O1—H1	0.8200	C3—H3	0.9300
O2—H2	0.8200	C4—C5	1.380 (2)
C1—O2	1.430 (2)	C4—H4	0.9300
C1—C2	1.498 (2)	C5—O1	1.371 (2)
C1—H1A	0.9700	C5—C6	1.391 (2)
C1—H1B	0.9700	C6—C7	1.380 (3)
C2—C7	1.388 (3)	C6—H6	0.9300
C2—C3	1.391 (2)	C7—H7	0.9300
C3—C4	1.388 (2)		
			
C5—O1—H1	109.5	C2—C3—H3	119.4
C1—O2—H2	109.5	C5—C4—C3	119.76 (15)
O2—C1—C2	109.44 (13)	C5—C4—H4	120.1
O2—C1—H1A	109.8	C3—C4—H4	120.1
C2—C1—H1A	109.8	O1—C5—C4	123.00 (14)
O2—C1—H1B	109.8	O1—C5—C6	117.03 (16)
C2—C1—H1B	109.8	C4—C5—C6	119.96 (16)
H1A—C1—H1B	108.2	C7—C6—C5	119.52 (17)
C7—C2—C3	117.92 (16)	C7—C6—H6	120.2
C7—C2—C1	120.83 (15)	C5—C6—H6	120.2
C3—C2—C1	121.25 (16)	C6—C7—C2	121.61 (16)
C4—C3—C2	121.22 (17)	C6—C7—H7	119.2
C4—C3—H3	119.4	C2—C7—H7	119.2
			
O2—C1—C2—C7	68.9 (2)	C3—C4—C5—C6	−0.6 (2)
O2—C1—C2—C3	−110.49 (18)	O1—C5—C6—C7	−179.38 (16)
C7—C2—C3—C4	−0.2 (2)	C4—C5—C6—C7	0.4 (3)
C1—C2—C3—C4	179.21 (16)	C5—C6—C7—C2	−0.2 (3)
C2—C3—C4—C5	0.5 (2)	C3—C2—C7—C6	0.1 (3)
C3—C4—C5—O1	179.21 (15)	C1—C2—C7—C6	−179.37 (16)

### Hydrogen-bond geometry (Å, °)

**Table:**

*D*—H···*A*	*D*—H	H···*A*	*D*···*A*	*D*—H···*A*
O1—H1···O2^i^	0.82	1.86	2.668 (3)	169
O2—H2···O1^ii^	0.82	2.01	2.817 (3)	167
C1—H1B···Cg1^iii^	0.97	2.77	3.694 (3)	159

Symmetry codes: (i) −*x*+3/2, *y*+1/2, *z*+1/2; (ii) *x*+1/2, −*y*+1/2, *z*−1; (iii) −*x*+1/2, *y*+1/2, *z*+1/2.
